# Assessment of Lipid Balance Parameters after Laparoscopic Sleeve Gastrectomy in 1-Year Observation

**DOI:** 10.3390/jcm12124079

**Published:** 2023-06-16

**Authors:** Patrycja Pawłuszewicz, Paweł Andrzej Wojciak, Aleksander Łukaszewicz, Jan Chilmonczyk, Jerzy Robert Ładny, Klaudiusz Nadolny, Hady Razak Hady

**Affiliations:** 1 1st Department of General and Endocrine Surgery, Medical University of Bialystok, 15-276 Bialystok, Polandladnyjr@wp.pl (J.R.Ł.); hadyrazakh@wp.pl (H.R.H.); 2 Surgical Ward, General Hospital, 18-200 Wysokie Mazowieckie, Poland; 3 Department of Emergency Medicine, Medical University of Bialystok, 15-089 Bialystok, Poland; 4 Faculty of Medicine, Katowice School of Technology, 40-555 Katowice, Poland; klaudiusz.nadolny@wst.pl

**Keywords:** obesity, sleeve gastrectomy, atherogenic dyslipidemia, lipid metabolism

## Abstract

**Introduction**: Currently, the increase in the percentage of obese people observed along with the development of civilization, reaching the level of a global pandemic, has forced a search for methods of effective and permanent obesity treatment. Obesity is a multifactorial disease; it coexists with many disease entities and requires multidisciplinary treatment. Obesity leads to metabolic changes in the form of metabolic syndromes, which include, among others, atherogenic dyslipidemia. The proven relationship between dyslipidemia and cardiovascular risk enforces the need to effectively improve the lipid profile of obese patients. Laparoscopic sleeve gastrectomy is a method of surgical treatment of morbid obesity which improves bariatric and metabolic parameters. The aim of the study was to assess the effectiveness of laparoscopic sleeve gastrectomy (LSG) at improving lipid profile parameters upon a 1-year follow up. **Material and Methods**: Bariatric parameters of 196 patients who underwent laparoscopic sleeve gastrectomy as well as the lipid profile of total cholesterol (TC), high-density lipoprotein (HDL), low-density lipoprotein (LDL), non-NDL, and triglycerides (TG) in a 1-year observation were analyzed. **Results**: Improvements in bariatric parameters were observed in patients after LSG. Total cholesterol, low-density lipoprotein (LDL), triglycerides and non-HDL level decreases were observed along with an increase in high-density lipoprotein (HDL) cholesterol levels. **Conclusions**: Sleeve gastrectomy is an effective method of treating obesity and improving the lipid profile in obese patients.

## 1. Introduction

Currently, the observed increase in the incidence of obesity is caused by global development and progress in all areas of civilization, in particular economic, industrial, infrastructure, social and cultural. The prevalence of obesity in the last twenty years has reached the level of a global pandemic, has made it one of the main issues and directions of the activities of all world public health organizations and is treated as a civilization disease, with huge socioeconomic and psychosocial importance [1]. Many years of research on obesity revealed its etiological and pathogenetic connection with many common diseases, such as cardiovascular disease, including atherosclerosis, hypertension, insulin-dependent diabetes, dyslipidemia, obstructive sleep apnea, bone and joint disease, depression, some types of cancer, reproductive system diseases and others [2,3].

The understanding of the relationship between obesity and other frequent metabolic co-morbidities has led to the definition of a new disease entity—metabolic syndrome. Currently applicable criteria for recognizing metabolic syndrome adopted by the International Diabetes Federation (IDF) and American Heart Association/National Heart, Lung and Blood Institute (AHA//NHLBI) in 2009 include abnormal waist circumference (depending on the population); abnormal triglyceride concentration (>150 mg/dL or the use of hypolipular treatment; abnormal HDL cholesterol fraction (<40 mg/dL (M), <50 mg/dL (K) or hypolipemic treatment used); abnormal arterial pressure (≥130/85 mm Hg or hypotensive treatment used); fasting glycemia (≥100 mg/dL) or use of hypoglycemic treatment. To recognize metabolic syndrome, the presence of three out of the five criteria [4] should be determined.

Analysis of the issues of pathological obesity and metabolic syndrome in terms of co-morbidity leads to the conclusion that they are a significant factor promoting the development of cardiovascular diseases, increasing the risk of the occurrence of myocardial infarction 2.5-fold, causing a 1.5-fold increase in total mortality and 2-fold increase in the frequency of all cardiovascular incidents, including brain stroke [5]. The proven fact is that the prevalence of this syndrome in the world population is increasing and reaches levels that allow us to treat it as a problem and disease threatening public health and civilization [6]. Metabolic syndrome occurs in a world population with a frequency of about 20 to even 40%, depending on the analyzed region and/or ethnic group and the age of patients. The occurrence of MS has a tendency to grow over time and gives disturbing prognoses regarding the frequency of its occurrence in the future; it is more common in women than in men, and its frequency increases significantly with age [7].

Lipid balance disorders are one of the main diseases coexisting with obesity and metabolic syndrome. Patients with abdominal obesity are more highly exposed to atherogenic dyslipidemia, which is associated with an increased risk of cardiovascular diseases with atherosclerotic disease and an increased risk of morbidity and mortality due to cardiovascular disease [8,9]. Research and treatment focus on improving lipid profiles in the pursuit of a potential reduction in diseases related to the cardiovascular system. To diagnose dyslipidemia in metabolic syndrome, practitioners are required to find disorder in triglyceride and lipoprotein concentrations in patient plasma: a TG concentration of ≥150 mg/dL and/or HDL concentration of <40 mg/dL (m) and <50 mg/dL (k). However, in the course of obesity and metabolic syndrome, deviations in the concentration and functions of other lipids are also observed, such as an increase in the concentration of very low lipoproteins of VLDL and chylomikrone, as well as of LDL.

The pathomechanism of the development of atherogenic dyslipidemia in metabolic syndrome is closely associated with insulin resistance and an excess of free fatty acids in the bloodstream. The accumulation of free fatty acids is the result of, among others, an intensified course of insulin resistance, lipolysis and their lowered uptake at the level of adipocytes. The excess of free fatty acids results in their accumulation in the liver, re-estrification into triglycerides and the intensified synthesis of TG-rich lipoprotein molecules with very-low-density VLDL [10]. At the same time, the influx of lipids supplied through food is higher in the case of insulin resistance than that in healthy subjects, which results in the phenomenon of postprandial hyperlipidemia. Exogenous lipids enter the bloodstream in the form of chylomikrone molecules, produced in enterocytes. Postable hyperlipidemia is also closely related to changes in the course of insulin resistance, because chylomikrons and VLDL are used in the same metabolic pathways [11].

The third factor increasing triglyceridemia is hepatic lipogenesis de novo, which is not inhibited in the case of insulin resistance and results in TG [12] increases. Hypertriglyceridemia associated with a high concentration of VLDL-1 stimulates adverse, biochemical changes in HDL and LDL lipoproteins. The process of replacing cholesterol esters with HDL and LDL for triglycerides from VLDL takes place through the cholesteryl ester transfer protein (CETP). As a result, the amount of cholesterol esters increases in VLDL, and the number of triglycerides increases in HDL and LDL. Furthermore, in the liver through the hepatic lipase, “small and dense” HDL and LDL [13] are created from these overloaded TG lipoproteins. Structurally abnormal HDL loses the possibility of cholesterol return transport from tissues (including blood vessels) to the liver, and also has a high plasma clearance, which is why it is quickly removed from the bloodstream and its concentration decreases. Small and dense LDL (SDLDL), on the other hand, has less affinity for receptors on hepatocytes than does normal LDL, which causes its longer maintenance in plasma [10].

## 2. Material and Methods

The aim of the study was to assess the effectiveness of LSG in the treatment of obesity, weight loss based on BMI, %TWL, %EWL, %EBMI examined after 1, 3, 6 and 12 months of observation and the assessment of LSG effectiveness in the treatment of dyslipidemia based on a change in total cholesterol, LDL, HDL, non-HDL and triglyceride concentrations.

Demographic, biometric and clinical data on patients from the study group were obtained prospectively on the day of surgery and as part of outpatient postoperative visits, which patients attended 1 month, 3 months, 6 months and 1 year after LSG. During these visits, blood samples were also taken in order to analyze laboratory and biochemical parameters.

The study group included 196 patients who underwent laparoscopic sleeve gastrectomy in our Department in 2016–2020 with complete data gathered during 12-month postoperative observation. A total of 107 men (54.6%) and 89 women (45.4%) were included in the study. The average age of patients in the study group was 44.9 years; the youngest patient was 21 years old, and the oldest was 66 years old. The median BMI value on the day of the operation was 47.7 kg/m^2^. A total of 30 (15.3%) patients reported hypercholesterolaemia as a comorbidity upon preoperative examination. In total, 43 (21.9%) patients were taking hypolipidemic drugs before surgery.

In terms of the statistical analysis, collected data were analyzed using STATA 13.0 software. Measurable variables observed in the study group for individual parameters subjected to statistical analysis are presented as averages with standard deviations (SD) or as a medians with interquartile intervals, according to each case. Statistical comparisons of measurable variables were conducted using repetitive measurements with a Wilcoxon test with multiple comparisons of post hoc variables. Pearson’s coefficient was used to evaluate the correlation of variables. Analyzed variables were considered statistically significant at the level of significance of *p* ≤ 0.05.

## 3. Results

The weight reduction rate as a result of LSG, analyzed on the basis of changes in bariatric parameters in the intervals of postoperative observation, is presented in Table 1. The results of body weight reduction analysis observed after laparoscopic sleeve gastrectomy are also reported. At the end of the 1-year follow up, the average BMI in the study group was 33.7 kg/m^2^.

The dynamics of weight loss in the group were similar in the observation intervals between 0 and 1 month, 1 and 3 months and 3 and 6 months, while in the interval between 6 and 12 months it slightly decelerated. Throughout the postoperative observation, a positive trend was maintained for body weight reduction, which was expressed as a negative trend of mean BMI values and positive trends of increases in the values of %TWL, %EWL and %EBL. Operated patients within 1 year after the procedure lost an average of about 1/3 of their total, initial body weight, which accounted for about 66% of their excess BMI resulting from obesity.

Furthermore, the plasma lipidogram was analyzed. For this purpose, repetitive determinations were conducted on total cholesterol (TC), triglycerides (TG), high-density lipoprotein (HDL-C), low-density lipoprotein (LDL-C) and the level of all lipoproteins associated with AP-B (non-HDL = TC-HDL-C) during the follow-up visits of patients in the 1st, 3rd, 6th and 12th month after laparoscopic sleeve surgery.

Before operation, only 30 (15.3%) patients reported dyslipidemia as a comorbidity but 86 (43.9%) patients had a HDL concentration of < 50 mg/dL (M) or a HDL concentration of < 40 mg/dL (K) and 112 (57.1%) had a triglyceride concentration of >150 mg/dL.

The final determinations of the plasma lipid profile of patients undergoing LSG surgery after 1 year from the procedure showed an average TC concentration of 175.9 mg/dL, so it decreased by 16.6 mg/dL in relation to the preoperative value. The level of the LDL-C fraction finally reached an average of 109.3 mg/dL, i.e., it was 14.5 mg/dL lower than the preoperative value.

**Figure 1:**
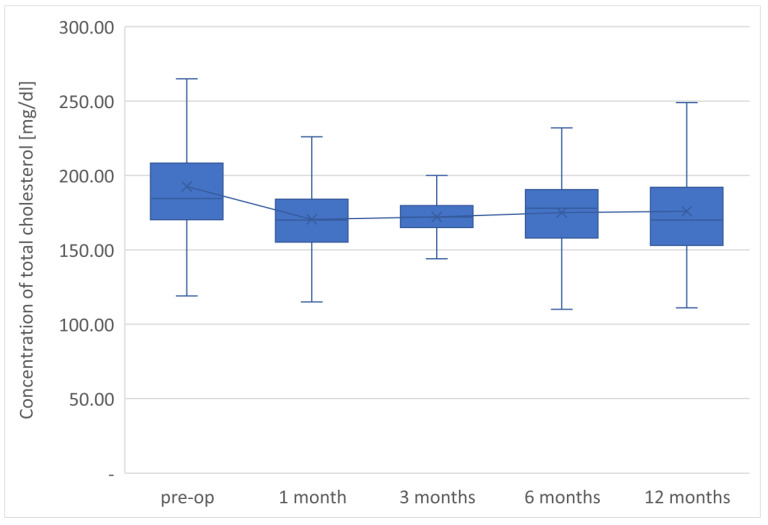
Changes in concentration of total cholesterol after LSG upon 12-month follow up.

**Figure 2:**
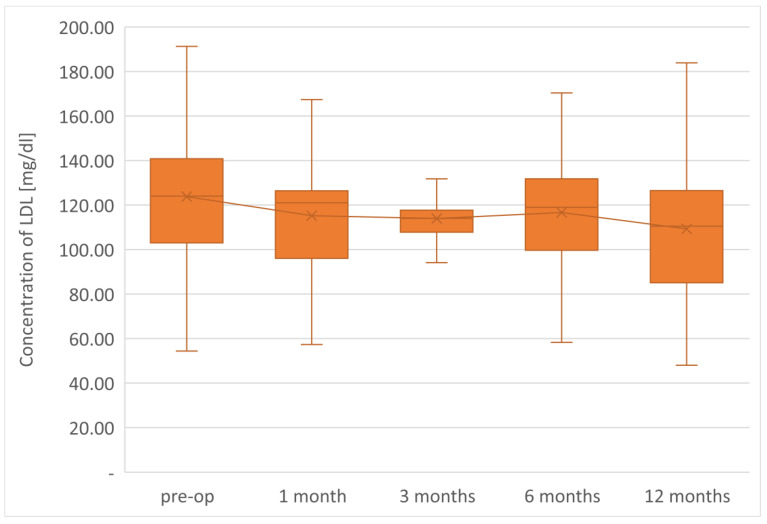
Changes in concentration of LDL after LSG upon 12-month follow up.

**Figure 3:**
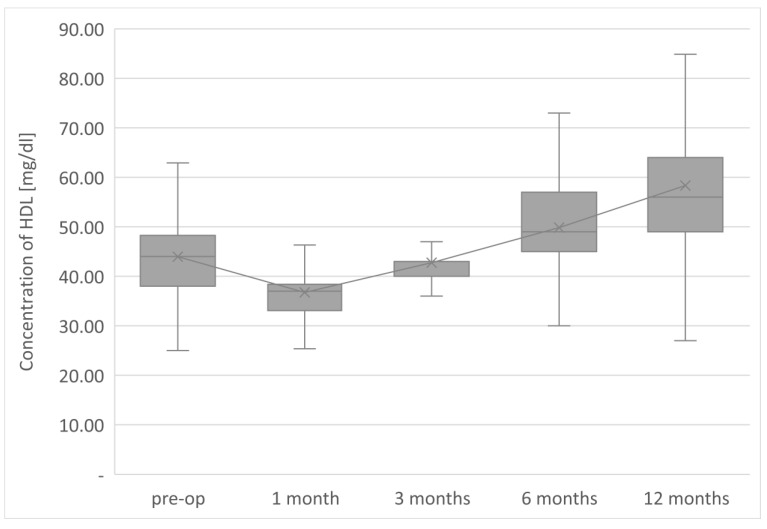
Changes in concentration of HDL after LSG upon 12-month follow up.

**Figure 4:**
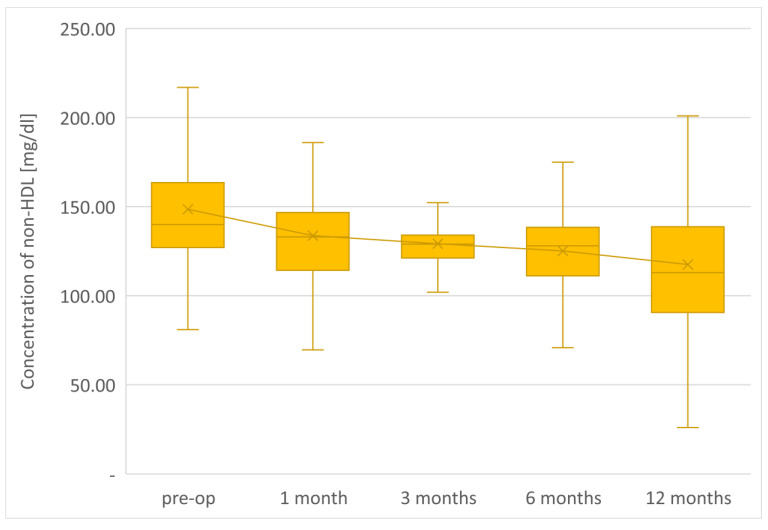
Changes in concentration of non-HDL after LSG upon 12-month follow up.

**Figure 5:**
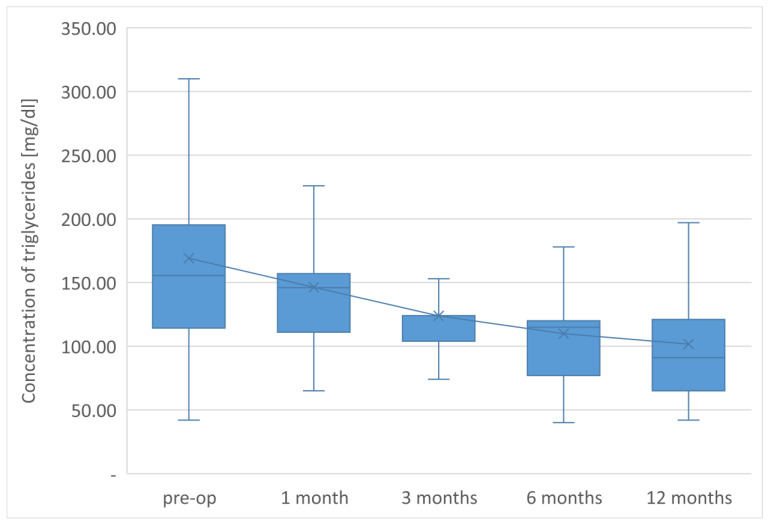
Changes in concentration of triglycerides after LSG upon 12-month follow up.

The average TG concentration in plasma significantly decreased from 148.6 mg/dL to 117.5 mg/dL. However, the average concentration of high-density lipoprotein fraction increased to 58.3 mg/dL, and at the end of the observation it was 14.3 mg/dL higher than that before LSG. The level of non-HDL lipoproteins in the plasma of examined patients at the end of the observation was 117.5 mg/dL and it was lower than the initial value by 31.1 mg/dL. All changes observed in this group of patients in the lipid balance parameters after 12 months were statistically significant (*p* < 0.05). The aforementioned changes in the lipid profile of patients after LSG are presented in Table 2 and Figure 1, Figure 2, Figure 3, Figure 4 and Figure 5.

The correlation between changes in concentrations of total cholesterol and LDL, HDL, non-HDL and triglycerides with the bariatric results obtained after 12 months after the surgery, in terms of BMI, %EWL, %EBL, %EBL, %TWL, was examined (Table 3). No correlation was observed between the bariatric results and the change in total cholesterol, low-density lipoproteins (LDL), non-HDL, or triglycerides. A correlation between BMI, %EWL and %EBL 12 months after surgery and a change in high-density lipoprotein concentration were demonstrated. *p* < 0.05 was recognized as the level of statistical significance.

## 4. Discussion

Obesity is undoubtedly one of the largest problems of modern health care. According to the World Obesity Federation, a population of 1 billion will live with obesity in 2030 [14]. The scale of the problem forces us to look for effective methods of preventing and treating obesity and coexisting diseases to reduce the morbidity and mortality associated with obesity.

The presented results of the 1-year observation of patients after sleeve gastrectomy confirm the effectiveness of surgery in the treatment of obesity and weight reduction. Considering the confirmed close relationship of body mass and obesity with comorbidity and mortality, statistically significant decreases in body weight and BMI after surgery lead to a conclusion of the indisputable health benefits after laparoscopic sleeve gastrectomy [14,15]. The results of available review and research publications analyzing the subject of postoperative weight loss, BMI, %TWL, %EWL, and %EBL after LSG were in line with the results obtained in our study [16,17]. Sleeve gastectomy is considered an effective and safe method of treating obesity [18,19].

Dyslipidemia is an asymptomatic disease. Patients are unaware of the disease. Dyslipidemia can be diagnosed only via laboratory tests, and a large part of the population does not report it. This delays proper treatment. Dyslipidemia should be treated in many ways, including through diet, physical activity, weight reduction and pharmacological agents. Patients applying for bariatric surgery due to obesity often do not report that they suffer from dyslipidemia, and it is diagnosed only after preoperative examinations. Only 15.3% of patients knew about the disease before surgery, only 21.9% were taking lipid-lowering drugs and more than 50% should have been diagnosed and treated dyslipidemia.

The parameters of the assessment of changes in a patient’s lipid profile after surgery were observed at the control points to be TC, TG, HDL, LDL and non-HDL lipid fractions. Analysis of changes in the above lipidogram parameters among patients undergoing laparoscopic sleeve gastrectomy after 1, 3, 6 and 12 months of postoperative control showed different, non-linear variations of the average concentrations of individual fractions in the group; however, the final effect of the procedure obtained after 1 year of observation was a reduction in the average atherogenic levels of lipids, i.e., TC, TG, LDL-C and non-HDL, which was accompanied by a simultaneous increase in the level of anti-atherosclerotic HDL-C lipoprotein.

Such clinically beneficial characteristics of changes in the lipid profile occurring after bariatric procedures is also described by many other authors studying the issue [20,21,22]. Regarding the average concentration of HDL-C lipid fractions and triglycerides in the plasma of analyzed patients obtained at the end of the observation and the criteria for diagnosing metabolic syndrome, it should be noticed that a decrease in the TG level below the threshold value of 150 mg/dL was obtained; however, the concentration of HDL-C increased above the desired amount of 50 mg/dL, so this procedure proved to be effective in the treatment of dyslipidemia defined by the criteria for metabolic syndrome. Our study proves the effectiveness of laparoscopic sleeve gastrectomy not only in weight reduction, but also in improving lipid metabolism.

An alternative non-invasive bariatric method is endoscopic sleeve gastroplasty. With endoscopic access, the volume of the stomach is reduced, and the mechanism of gastric emptying is also impaired. It leads to an earlier feeling of satiety, reduced food intake and weight loss. Its advantage over laparoscopic sleeve gastrectomy is the lack of scars and the possibility of reversing anatomical changes. The procedure can be performed under intravenous anesthesia, which is an alternative for patients who are not eligible for surgical treatment. Studies confirm the effectiveness of endoscopic sleeve gastroplasty in the treatment of metabolic disorders and weight reduction in obese patients [23].

In the assessment of cardiovascular risk, researchers and clinical practitioners take into account the treatment of atherogenic dyslipidemia, based mainly on the reduction in the low-density fraction of LDL cholesterol. Scientific research confirms that a reduction in the LDL fraction correlates most closely with a reduction in cardiovascular risk [24]. Scientific data are available that indicate the role of non-HDL as an alternative therapeutic determinant in the assessment of CVD risk, covering all fractions containing apoB with a stronger correlation with CDV risk than LDL concentration in patients with a high TG concentration who were not fasting at the time of examination. Non-HDL may be considered an alternative therapeutic goal in the treatment of dyslipidemia and prevention of cardiovascular diseases, but it is not widely used [25]. In connection with the above, a beneficial effect of bariatric operations may be observed in CVD risk reduction based on changes in the lipid profile in the form of a decrease in LDL.

The atherogenic properties of lipoproteins rich in TG are widely proven [26]. Some authors indicate the significant importance of hypertriglyceridemia and low HDL levels in the increase in cardiovascular risk. Although genetic evidence indicated triglycerides (TG) in plasma as an independent risk factor of CVD, no consensus has been reached as to the targeting of the elevated level of these lipoproteins to prevent CVD [26].

## 5. Conclusions

Laparoscopic sleeve gastrectomy is an effective method of morbid obesity treatment.

Sleeve gastrectomy improves lipid profiles independently of weight loss, reducing the concentration of total cholesterol, LDL, non-HDL, and triglycerides and increasing HDL concentration. The therapeutic effect is observed in lipid fractions, the concentrations of which are taken into account in the diagnosis of MS.

The correlation of bariatric effects with a change in high-density lipoproteins was proven. No such correlations were observed in other lipid fractions.

## Figures and Tables

**Table 1:** Changes in bariatric parameters in 1-year follow-up.

	Before the Surgery	1 Month		3 Months		6 Months		12 Months	
		Mean		Mean		Mean		Mean	
Body mass (kg)	145.7 ± 25.0	129.3 ± 22.7 *		117.4 ± 21.1 *		106.5 ± 20.8 *		101.4 ± 20.6 *	
BMI (kg/m^2^)	48.3 ± 6.8	42.9 ± 6.3 *		39.0 ± 6.1 *		35.4 ± 6.0 *		33.7 ± 6.0 *	
%EBL		24.3 ± 6.6 *		42.2 ± 10.7 *		58.4 ± 14.8 *		66.0 ± 17.5 *	
%EWL		21.6 ± 5.5 *		37.5 ± 8.8 *		51.9 ± 12.2 *		58.7 ± 14.6 *	
%TWL		11.2 ± 2.5 *		19.5 ± 3.6 *		27.0 ± 5.2 *		30.5 ± 6.3 *	

* *p* < 0.05—statistically significant.

**Table 2:** Changes of concentration of total cholesterol, LDL, HDL, non-HDL and TG.

	Before the Surgery	1 Month	3 Months	6 Months	12 Months
TC [mg/dL]	192.5 ± 37.8	170.5 ± 36.2 *	172.1 ± 28.8 *	175.0 ± 30.5 *	175.9 ± 34.0 *
LDL [mg/dL]	123.8 ± 31.2	115.2 ± 32.3 *	114.0 ± 25.9 *	116.7 ± 29.2 *	109.3 ± 33.0 *
HDL [mg/dL]	44.0 ± 8.7	36.8 ± 6.9 *	43.0 ± 7.5 *	49.9 ± 10.6 *	58.3 ± 15.5 *
Non-HDL [mg/dL]	148.6 ± 37.8	133.7 ± 36.5 *	129.2 ± 28.2 *	125.2 ± 31.1 *	117.5 ± 35.4 *
TG [mg/dL]	169.1 ± 94.9	146.3 ± 50.8 *	123.8 ± 40.1 *	109.9 ± 43.8 *	101.7 ± 47.5 *

* *p* < 0.05—statistically significant.

**Table 3:** Correlations between changes in concentrations of total cholesterol, LDL, HDL, non-HDL and triglycerides with changes in bariatric parameters after 1-year after LSG.

	∆TC	∆LDL	∆HDL	∆non-HDL	∆TG
BMI	R^2^ = 0.0001 *p* = 0.8793	R^2^ = 0.0022 *p* = 0.5171	R^2^ = 0.218 *p* = 0.0389	R^2^ = 0.019 *p* = 0.5482	R^2^ = 0.0069 *p* = 0.2485
%EBL	R^2^ = 0.0024 *p* = 0.4987	R^2^ = 0.0006 *p* = 0.7329	R^2^ = 0.0245 *p* = 0.0284	R^2^ = 0.0001 *p* = 0.8919	R^2^ = 0.0000 *p* = 0.9414
%EWL	R^2^ = 0.0028 *p* = 0.4622	R^2^ = 0.0003 *p* = 0.8081	R^2^ = 0.0213 *p* = 0.0413	R^2^ = 0.0000 *p* = 0.9798	R^2^ = 0.0003 *p* = 0.8132
%TWL	R^2^ = 0.0045 *p* = 0.3496	R^2^ = 0.0010 *p* = 0.6661	R^2^ = 0.0073 *p* = 0.2352	R^2^ = 0.0012 *p* = 0.6340	R^2^ = 0.0112 *p* = 0.1405

## Data Availability

Not applicable.
